# Translational research in kidney transplantation and the role of patient engagement

**DOI:** 10.1186/s40697-015-0077-2

**Published:** 2015-11-05

**Authors:** Janine F. Farragher, Meghan J. Elliott, Samuel A. Silver, Zsuzsanna Lichner, Anne Tsampalieros

**Affiliations:** Division of Nephrology, University Health Network, Toronto ON, Canada and the Rehabilitation Sciences Institute, University of Toronto, Toronto, ON Canada; Li Ka Shing Knowledge Institute, St. Michael’s Hospital, Toronto, ON Canada; Division of Nephrology, St. Michael’s Hospital, University of Toronto, Toronto, ON Canada; Department of Laboratory Medicine and the Keenan Research Centre for Biomedical Science at the Li Ka Shing Knowledge Institute, Toronto, Canada; Division of Nephrology, Children’s Hospital of Eastern Ontario, Clinical Epidemiology Program and the University of Ottawa, Ottawa, ON K1H 8 L1 Canada

## Abstract

**Background:**

Translational research is an evolving discipline that is intended to bridge the gaps between basic science research, clinical research, and implementation in clinical practice. It is a fluid, multidirectional process that requires strong interdisciplinary collaboration to produce research that is relevant to end-users.

**Purpose of this review:**

This review summarizes current perspectives on translational research and outlines its relevance and importance to kidney transplantation research.

**Sources of information:**

Sources of information used for this review include published reports, articles, and research funding websites.

**Findings:**

Tissue typing is used as an in-depth example of how translational research has been applied in the field of kidney transplant medicine, and how it has resulted in successful implementation of diagnostic and management options for sensitized individuals undergoing kidney transplantation. The value of actively involving kidney transplant stakeholders (patients, caregivers, and clinicians) in setting research priorities and determining relevant outcomes for future investigation is also discussed.

**Limitations:**

This is a narrative review of the literature which has been partly influenced by the perspectives and experiences of its authors.

**Implications:**

Translational and patient-oriented research practices should be incorporated into future research endeavours in the field of kidney transplantation in order to create beneficial change in clinical practice and improve patient outcomes.

**What was known before:**

Translational research which engages patients in the investigative process can enhance the likelihood that medical discoveries will have a meaningful impact at the bedside.

**What this adds:**

This article applies current perspectives on translational research and patient engagement to the field of kidney transplantation, illustrating how these approaches have led to significant advancements in the field. It provides further justification for deliberate, targeted efforts to cross-collaborate and incorporate the patient voice into kidney transplant research.

## Abrégé

**Contexte:**

La recherche translationnelle est une discipline évolutive qui a pour but de faire le pont entre la recherche fondamentale, la recherche clinique et la mise en œuvre de pratiques cliniques dans le domaine des transplantations rénales. Il s’agit d’un processus multidirectionnel et fluide qui demande la collaboration étroite de toutes les disciplines impliquées afin que la recherche qui en résulte soit pertinente et touche directement les usagers.

**Objectifs de la revue:**

Cette revue fait la synthèse des éléments actuels de la recherche translationnelle, et décrit sa pertinence et son importance dans le domaine de la recherche sur la transplantation rénale.

**Sources:**

La réalisation de cette revue a été possible suite à la consultation de recueils et d’articles publiés ainsi que de sites web dédiés au financement de la recherche.

**Constatations:**

L’étude de la compatibilité immunologique est utilisée à titre d’exemple pour démontrer en détail la façon dont la recherche translationnelle a été appliquée dans le domaine des greffes du rein jusqu’à maintenant, et comment elle a permis la mise en œuvre de solutions efficaces pour le diagnostic et l’organisation des soins aux patients subissant une greffe de rein. On a également discuté de l’importance d’impliquer toutes les parties prenantes d’une procédure de transplantation rénale, soit les patients eux-mêmes et le personnel soignant et le personnel clinique, afin d’établir les priorités de recherche et de définir les résultats pertinents en vue d’études ultérieures.

**Limites de l’étude:**

Il s’agit d’une revue non systématique de la littérature influencée en partie par la perspective et les connaissances des auteurs sur le sujet.

**Conséquences:**

Il apparait important d’intégrer les pratiques courantes en recherche translationnelle de même qu’en recherche axée sur le patient lors de futures études sur les greffes de reins. Ceci afin d’instaurer un changement bénéfique dans la pratique clinique et par conséquent, d’améliorer les résultats chez les patients.

**Données connues:**

Une approche de recherche translationnelle favorisant l’implication des patients dans le processus d’analyse peut augmenter les chances de voir les découvertes médicales avoir des répercussions directes et plus significatives pour le patient.

**Ce que cette étude ajoute:**

Cette revue expose les différents points de vue sur la recherche translationnelle et la collaboration des patients au processus, dans le domaine de la transplantation rénale. Elle illustre également la façon dont ces approches ont mené à des progrès marqués dans le domaine et plaide pour une collaboration volontaire et ciblée entre les différents intervenants ainsi que pour une plus grande implication des patients dans la recherche.

## Why is this review important?

A kidney transplant is the best treatment for patients with end-stage renal disease. This review highlights the importance of translational research in bridging the gaps between basic and clinical research and promoting evidence implementation in the field of kidney transplantation. It also reviews the key role of patient engagement in the research process.

## What are the key messages?

The example of tissue typing is provided to illustrate the application of translational research in kidney transplantation. Patient-oriented research, including the involvement of kidney transplant stakeholders in determining research priorities and outcomes, may enhance the relevance and implementation of research findings into practice.

## Implications for future research/policy

Translational research fosters multidisciplinary and multi-stakeholder collaboration and can improve translation of findings into practice. This strategy lends itself to many opportunities to enhance patient care and quality of life post-kidney transplant.

## Introduction

A Medline search exploring the term “translational research” reveals titles published as early as the 1990s [1]. The discipline of translational research, however, has come into existence more recently [2]. The concept of translational research may have different meanings, depending on the field [3]. For researchers, it might imply testing an idea in a laboratory with the hopes of bringing the finding into a clinical setting [4], whereas for clinicians, it may imply seeing the benefits of laboratory discoveries at the bedside and changing practice guidelines [3, 5, 6].

The National Institutes of Health initially defined translational research as two separate areas of research: the first involving the application of discoveries from the laboratory or “bench” to the clinical setting, and the second aimed at adapting “best practices” in the community. It was thought to exist along a unidirectional continuum [7]. Over the years this definition has evolved to become bi-directional and to include more phases [1]. Waldman et al. describe a newer model which now reflects a more diverse spectrum of knowledge [8]. T1 phase translates basic laboratory research to human application [9]. T2 phase promotes the movement of research discoveries through clinical development in order to gather enough evidence to develop practice guidelines [10]. During the T3 phase, the findings made in T1 and T2 are brought to community practice [11]. This phase was included so that all patients could benefit from the discoveries made in the first two phases. The T4 phase aims to incorporate a public health model, with the goal being to educate the community to make healthier choices in order to prevent disease [9]. Waldman et al. also propose including a T0 and T5 phase. T0 recognizes the work of laboratory investigators as being the start of the continuum, whereas T5, at the other end of the translational research spectrum, allows for a more “global” approach by extending research to a societal model [12].

**Fig. 1:**
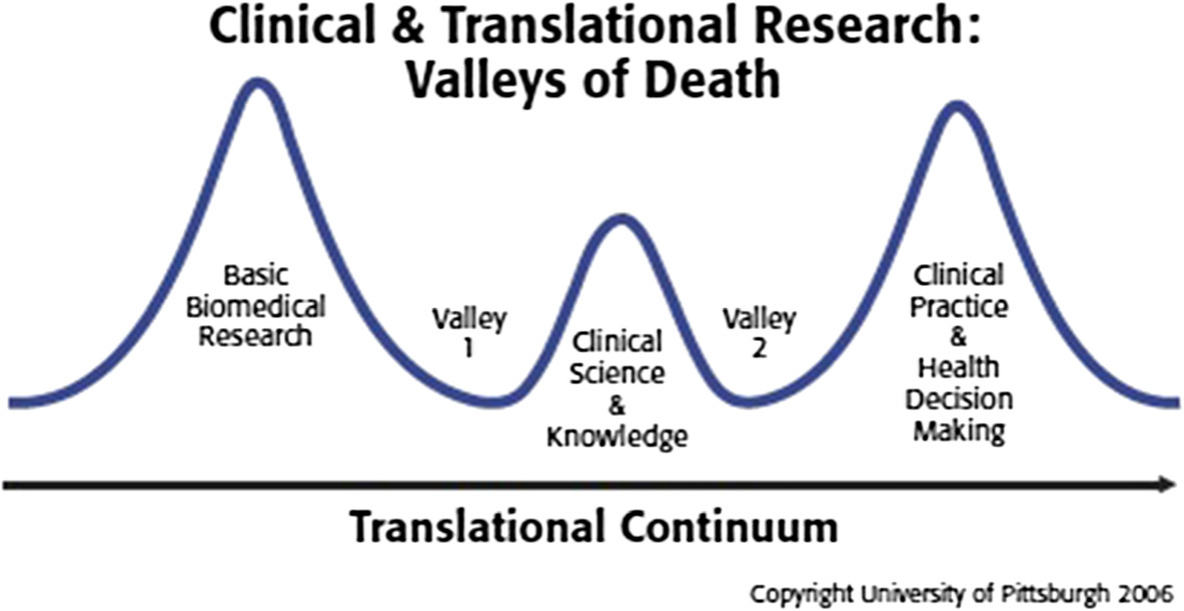
Valleys of Death in Translational Research. This figure illustrates the “death valleys” which have been described by the Canadian Institutes of Health Research. The proposed two valleys can occur between the 3 phases of translational research. The first occurs in translating results from the laboratory to the bedside, and the second in attempting to translate knowledge to health-making decisions. Adapted from figure 1 [15] with permission from Wiley

Some of the benefits of translational research include an increased number of individuals participating in research and a more patient-centered approach [2]. Obstacles include the high cost of the desired projects, a lack of funding, and slow turnover of results [3, 13]. The Canadian Institutes of Health Research (CIHR) refers to the challenges Canada faces in promoting translational research as “death valleys” [14, 15] (Fig. 1). Valley 1 refers to “*the decreased capacity to translate the results of discoveries generated by basic biomedical research in the laboratory to the bedside or careside …*”, and Valley 2 refers to the “*limited capacity to synthesize, disseminate and integrate research results more broadly into health care decision-making and clinical practice*” [14]. In this narrative review, we will highlight examples of translational research and the death valleys within the field of kidney transplantation, and review tissue typing as an in-depth example of bench-to-bedside research. We will also discuss the role of patient-oriented research in translational research, and its use and applicability to the field of kidney transplantation.

### Biomarkers of acute rejection in kidney transplant recipients: challenges in bridging Valley 1

A kidney transplant is the preferred treatment for patients with end-stage renal disease (ESRD). Despite improvements in graft survival, long-term management post-transplant is still challenging. The constant risk of rejection and the long-term side effects of immunosuppressive medications remain obstacles. Early acute rejection affects 10 % of kidney transplants [16–18]. Measuring serial serum creatinine levels is one way of monitoring kidney function; however, a rise in creatinine level is a late sign of kidney dysfunction and cannot differentiate between possible causes. A kidney biopsy is the gold standard for diagnosis, but is both invasive and subject to sampling error [19]. The use of biomarkers as non-invasive diagnostic tools in kidney transplantation has been described since the 1970s [20]. A biomarker is a “*cellular, biochemical, molecular or genetic alteration by which a biological process can be recognized and/or monitored and has diagnostic or prognostic utility*” [21]. Urine is a potential source for biomarker monitoring of kidney function, including proteins, peptides and messenger RNAs [22].

Halawa reviews some of the biomarkers that have been considered in kidney transplantation in an attempt to detect kidney injury earlier [18]. One of the more extensively studied biomarkers is human neutrophil gelatinase associated lipocalin (NGAL). NGAL is normally expressed at low levels in kidneys and increases tremendously after kidney injury. NGAL has been measured in kidney biopsies [23], serum before and shortly after transplantation to predict delayed graft function [24], and urine samples to detect tubulitis [25]. Heyne et al. [26] measured NGAL from spot urines in 182 outpatient kidney transplant recipients in order to discriminate acute rejection from other causes of kidney dysfunction. They found that patients with biopsy-proven rejection had much higher NGAL levels, and the sensitivity and specificity to predict rejection were high at 100 % and 93 % respectively. A study by Blydt-Hansen et al. [27] assessed the utility of metabolomics in detecting T cell-mediated rejection among pediatric transplant recipients. This study demonstrated that urinary metabolomics are both sensitive and specific in detecting T cell-mediated rejection in this population. However, despite the many possible advantages of using biomarkers, their clinical utility remains unclear, and they are not currently part of routine clinical care in most centres. Some of the reasons for this include that they were developed in a non-transplant setting and thus still require validation in larger trials of transplant patients [18], or that they are not readily available at all centers. Furthermore, none of the studies to date have assessed whether measuring these biomarkers leads to improvement in clinical outcomes compared to the current management of kidney transplant recipients. These examples demonstrate some of the challenges seen with translational research, and in particular within Valley 1.

### Defining the optimal treatment for antibody-mediated rejection after kidney transplantation: Challenges in bridging Valley 2

Acute antibody-mediated rejection (ABMR) is another form of rejection that occurs in 5–7 % of transplants and causes 10–48 % of acute rejection episodes post-kidney transplant [28]. ABMR is less responsive to therapy, and one-year graft survival ranges between 15–50 % [29]. Current international guidelines do not have a defined evidence-based treatment protocol for ABMR, and the Kidney Disease Improving Global Outcomes (KDIGO) guidelines suggest the use of one or more of a variety of therapeutic modalities [30, 31]. Sureshkumar et al. [30] reviewed six studies that tested therapies for the treatment of ABMR, including the use of steroids, plasmapheresis, intravenous immunoglobulin, and/or monoclonal antibodies. Most of these studies demonstrated improvements in graft function after treatment compared to their control arm. A more recent systematic review by Roberts et al. [32] identified 12 controlled trials (five randomized and seven non-randomized) that compared the efficacy of therapies used for acute ABMR post-kidney transplant. The included studies were quite heterogeneous, and the review concluded “*there is currently insufficient evidence to guide treatment for acute ABMR”.* This area of research highlights an example of Valley 2, as the clinical studies that have been performed in this area lack the size and quality to provide compelling evidence needed to make standardized practice recommendations.

### An in-depth example of the translational research process: tissue typing

The knowledge sharing and collaboration that has occurred across the valleys in the field of tissue typing exemplify how translational research has led to improved patient outcomes. This process began with a series of early discoveries in the laboratory. In the 1930s, P.A. Gorer, a physician, studied a possible link between blood group antigens and rejection of allogeneic tumor transplants in mice and observed that the rejection reaction of tumor grafts resembled the reaction to incompatible blood transfusions. Meanwhile, G. Snell, a geneticist, studied the genetics of the histocompatibility loci. The two established that the blood group antigens and the histocompatibility H locus were the same, and termed the locus H2 [33]. Jean Dausset observed a correlation between leukocyte antigen compatibility and skin graft tolerance, and in 1965 he proved that the human leukocyte antigen (HLA) Hu-1 complex was a transplantation antigen. Baruj Benacerraf, working with guinea pigs, noticed that when injected with a synthetic antigen, only a subset of animals responded. Through a series of cross-matching experiments, he proved that the response was controlled by a single dominant gene, which he termed ‘immune response’ or Ir gene. The Ir gene turned out to be a member of the major histocompatibility complex (MHC). Working with genetically identical twin donors, Benacerraf proposed that rejection of transplanted organs is governed by Ir genes [34]. These findings paved the way for an understanding of autoimmune diseases, organ transplantation, and how individuals in a population respond to the same pathogen. HLA genes are the most polymorphic loci described to date in the human body. Today HLA typing forms the basis of donor selection. Despite its complexity, HLA typing has provided kidney transplant programs with the ability to specify a patient’s antibody profile, and has been translated into patient-centered transplant pathways.

The initial serological assays included complement-dependent cytotoxicity (CDC). CDC-based methods, where recipient serum is mixed with donor cells, were the first tests used to identify circulating antibodies [35]. Their implementation prior to every kidney transplant quickly reduced the incidence of hyperacute rejection. However, CDC methods could not define all antibody specificities, with several false negatives and false positives [35–37]. This was particularly a problem for sensitized patients, who were often denied a potential donor kidney based upon a false positive crossmatch [37, 38]. Therefore, the most vulnerable patients were not maximally benefiting from this new technology.

The sensitivity and specificity of histocompatibility testing needed to be improved to enable rapid translation to the entire transplant population. This process was partly facilitated by International Histocompatibility Workshops, which helped standardize research techniques to enable collaboration between different researchers and laboratories [39]. Improved immunoassays, such as the solid-phase enzyme-linked immunosorbent assay and bead-based methods (Luminex, FlowPRA), greatly increased sensitivity and specificity. CDC panel reactive antibody (PRA) assay is now based on flow cytometry and can be performed prior to and after transplantation (FlowPRA test). These advances allowed for the determination of unacceptable donor antigens by transplant programs [35, 37, 40].

While these advances improved the ability to describe a patient’s immunologic risk, solutions were still needed to translate better risk stratification into improved outcomes that matter to patients. This challenge was accepted by clinical and health service researchers, who could now properly assess the benefits and risks of innovative transplant options for sensitized patients. Three protocols for sensitized patients now exist: acceptable mismatch, kidney paired donation, and desensitization, with the choice based primarily on an individual patient’s antibody profile [41, 42].

The first two options take advantage of HLA technology to avoid donor specific antibodies that would elicit an immune response. Acceptable mismatch programs use large donor pools to identify HLA antigens toward which a recipient has never formed antibodies [38, 43, 44]. In this way, a sensitized patient may receive a kidney from a donor that contains these self-antigens and other closely related HLA antigens [38]. Despite their successes, acceptable mismatch programs require access to a large inventory of HLA typed cells or assays, and so are best suited for sensitized patients with common HLA phenotypes [38].

Patients with rare HLA phenotypes who are not suitable for acceptable mismatch programs may still find a donor through kidney paired exchange. Kidney paired donation programs match incompatible donor-recipient pairs to each other, leading to compatible pairs and avoidance of donor-specific antibodies [45]. These programs could not exist without the ability to consistently predict an acceptable match, and thus kidney paired donation is most likely to result in a match for patients with a relatively narrow breadth of sensitization, such as those with a single high-titre antibody to their original donor [42]. However, kidney paired exchange is dependent on both living donors and enrollment of a large number of donor-recipient pairs [46, 47].

Desensitization remains an option to enable transplantation of a mismatched kidney for those who cannot access a kidney in either of the previous programs [48–51]. Desensitization is best suited to broadly sensitized patients, since these patients are difficult to match in both acceptable mismatch and kidney paired donation programs [52]. HLA typing is once again critical to successful desensitization, since antibody titres are used to determine if desensitization is likely to be effective and when immunosuppression has sufficiently lowered antibody titres to facilitate safe transplantation of a mismatched kidney [38, 52].

In Canada, both a living donor paired exchange and highly sensitized patient program exist [53]. The latter is a national deceased kidney donor organ sharing agreement between provincial transplant programs that gives each program access to a larger number of potential donors for their highly sensitized patients. As of November 2014, all provinces have joined the program. Since the first provinces joined the program in October 2013, approximately 120 highly sensitized patients have received kidneys [54]. For Canadian patients who do not receive a kidney through these options, several centers have active desensitization programs. Results are typically better than dialysis [55], but outcome reporting is susceptible to selection bias and different antibody reporting practices between centers [56]. For these reasons, physicians interested in desensitization for a patient should discuss this possibility further with their local transplant center for information on feasibility and outcomes.

How did histocompatibility testing successfully translate from knowledge to practice and create several transplant options for the sensitized patient? This question is difficult to answer with certainty, but interdisciplinary collaboration appears to have played a major role. One excellent example is the Banff initiative, which is an ongoing interdisciplinary effort to standardize definitions of rejection involving elements from both histocompatibility and pathology [57, 58]. Patient-level innovations were needed beyond enhanced risk assessment, which is outside the expertise of many basic scientists. Meanwhile, accurate immunologic risk prediction and antibody classification seemed to be exactly what clinical and health service researchers required to translate their ideas into individualized care pathways for sensitized patients.

There are also several histocompatibility clinical observations that may be candidates for reverse translation from the bedside back to the bench. Firstly, solid phase assays sometimes identify donor specific antibodies despite a negative CDC crossmatch [36, 59], the significance of which requires clarification [60–64]. Secondly, donor specific antibodies can arise after transplantation, and it remains unclear what triggers their appearance and immunogenicity [65–68]. Thirdly, the highest antibody titre does not always correlate with end-organ damage, necessitating better techniques to predict the severity of immune responses [35, 38, 69].

The HLA story demonstrates how translational research is a fluid process that requires collaboration between basic scientists and clinical researchers. It describes one example of successful translational research in kidney transplantation, where basic science discoveries resulted in specialized treatment options depending on an individual patient’s antibody profile. As a consequence, kidney transplant patients can receive the transplant option most appropriate to their own circumstances, leading to better health outcomes at the population level.

### Patient-oriented research in kidney transplantation

One of the key barriers to bridging the research-to-practice valleys identified by the CIHR is the limited role of patients in research [14]. While the research agenda is typically driven by investigators, the primary end users of research are patients and the clinicians who care for them. Therefore, mismatches that occur between research foci and patient priorities can lead to frustration [70]. Patients living with a particular condition, such as kidney transplant recipients, bring expertise on that condition and its implications in daily living, so it follows that they should have a voice in establishing research priorities. Further, patient and public involvement in research has been found to positively impact all stages of the research process, from the development of user-relevant questions to user-focused implementation strategies [71].

Traditionally, approaches to defining research priorities have not involved patients, which can make it challenging to bridge either of the death valleys along the translational research continuum. Each year, up to $240 billion is spent funding biomedical research, of which up to 85 % is considered wasted [72]. This waste can occur at any stage in the production and reporting of research, including the failure to address relevant questions and to involve end users of research [73]. The importance of engaging key stakeholders in research prioritization has been emphasized by funding agencies, and has led to the development of national strategies and organizations that aim to engage patients in the research process. These include the Strategy for Patient-Oriented Research in Canada (supported by the CIHR) [14], the Patient-Centered Outcomes Research Institute in the United States (supported by the U.S. government) [74], and INVOLVE in the United Kingdom (supported by the National Institute for Health Research, U.K.) [75].

Few evidence-based methods for involving patients and other stakeholders in determining research priorities exist. The Cochrane Agenda and Priority Setting Methods Group has identified three such processes: the health equity lens model [76], the dialogue model [77], and the James Lind Alliance (JLA) priority setting partnership [78]. Of these, the JLA model is the most established method and has been used successfully to date in setting research priorities in more than 25 conditions. Although little work has been done in the area of stakeholder involvement in research prioritization in kidney disease, a recent study in patients with ESRD on or nearing dialysis and their clinicians and caregivers employed this technique to arrive at a list of top ten ESRD-related research uncertainties [79]. These included questions on enhancing communication, dietary restrictions, dialysis modality options, vascular access, and access to transplantation.

While studies on research prioritization in kidney disease in general provide valuable information that will help guide future research, patients who have received a kidney transplant represent a separate population with likely different concerns and priorities. Little work has been done on patient involvement in kidney transplant-related research prioritization. A recent systematic review evaluating approaches to research prioritization in kidney disease found only four studies that identified research priorities in kidney transplantation [80]. These four studies used diverse methods for identifying research priorities, and only two included patients in the prioritization process [79, 81]. Only one study looked at research prioritization specific to kidney transplantation in the pediatric population [82]. In these studies, questions of etiology, diagnosis, treatment, health services, and psychosocial issues as they relate to kidney transplantation were identified as priorities. While organizations and funders emphasize the importance of stakeholder engagement in research prioritization, some challenges and shortcomings of this approach must be acknowledged. The optimal method to engage patients and other stakeholders remains unclear, and few studies explicitly describe the prioritization processes used in detail [79, 80]. Further, these processes rely on subjective viewpoints of participants, whose perspectives, values and priorities can change over time and differ across contexts and populations [80]. Another shortcoming of patient-centered research is the occurrence of conflicts between different priorities. For instance, in an effort to avoid inequity in access to transplantation, lower weight is given to optimal HLA matching between donors and recipients in US organ allocation schemes, which may lead to reduced graft survival [83]. Future studies using established and explicit methods to engage kidney transplant recipients in determining transplant-related research priorities are needed to ensure that relevant research is conducted and translated into practice.

**Fig. 2:**
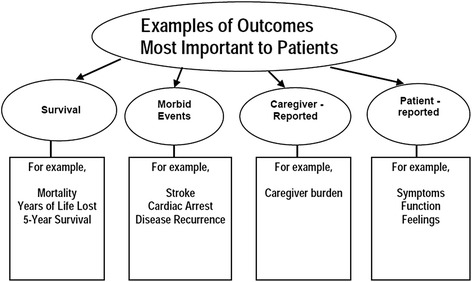
Summary of Important Outcomes to Patients. This figure outlines the various clinical trial outcomes that are considered to be important to patients. Adapted from figure 17.1.a [64] with permission from Wiley

In addition to involving patients in research priority setting, there has been a growing recognition of the importance of using patient-reported outcomes (PROs) to ensure treatments lead to meaningful health improvements for patients. PROs have been defined as “*reports coming directly from patients about how they feel or function in relation to a health condition and its therapy without interpretation by healthcare professionals or anyone else*” [84]. Not only are PROs themselves among the most important outcomes to patients (Fig. 2), they are also frequently associated with other outcomes such as morbidity and mortality. Examples of concepts included under the PRO umbrella are health-related quality of life (HRQoL), symptom burden, functional status, and beliefs, perceptions and experiences surrounding various aspects of treatment such as convenience and tolerability.

PROs have been used in kidney transplant research for various purposes. One salient example has been to address medication non-adherence among kidney transplant recipients. Medication non-adherence is a prevalent issue in kidney transplant patients [85], which is associated with an increased risk of mortality and graft rejection [86]. Using PROs, kidney transplant researchers have been able to elucidate some of the barriers to adherence among patients such as false beliefs about medications [87, 88] and forgetfulness [87–89], and also observe differences in side effects and quality of life associated with different medication regimens [90, 91] which may have an effect on adherence. The former information has helped to stimulate research into enhanced patient education and follow-up protocols [92, 93], which combined with related literature from other clinical populations has informed the Kidney Disease Improving Global Outcomes (KDIGO) clinical practice guidelines on addressing medication adherence in transplanted patients [31]. This is an example of how PROs and T2 research initiatives can collectively impact expert consensus on best practice. Future T3 research initiatives may seek to further promote, educate, and evaluate the use of these guidelines among practitioners, in order to maximize the real-world impact on patient outcomes.

Another example of PRO use is in the growing field of geriatric transplantation, where quality-of-life outcomes hold particular significance. Research has demonstrated that elderly transplanted patients experience better HRQoL than elderly patients on dialysis, but they also score more poorly than normative age-matched populations in some HRQoL-related domains such as functional status [94–97]. Older adults who demonstrate these forms of functional impairments often benefit from a more geriatric approach to care which may include active rehabilitation to improve strength, mobility, and adaptive living skills, and reduce the risk of falls [98–102]. Thus, future T2 research initiatives may build off this HRQoL data to investigate the potential benefits of similar care approaches for older adults with renal transplants.

Despite their demonstrated potential to lead to meaningful improvements in patient wellbeing, PROs currently occupy a small proportion of the outcome literature in kidney transplantation. For example, a systematic review which examined the use of PRO measures in immunosuppressive regimen trials concluded that very few randomized controlled trials had used HRQoL outcomes. Efforts to incorporate PROs into research have also often been minimized by various challenges - the same review found that when PROs such as HRQoL were included in such trials, the validity of the measures used or the clinical relevance of the results were often not considered [103]. PRO-focused research is also susceptible to the death valleys of translational research, which may be particularly true if there is no clearly established rationale or future implication(s) associated with assessing PROs. This has been demonstrated in clinical practice, where simply routinely assessing HRQoL, for example, has been found to have minimal observable impact on treatment [104]. Thus, engaging in full-spectrum translational PRO research should be an ongoing area of focus for transplant researchers, to maximize the likelihood that research evidence translates into meaningful improvements for patients.

## Conclusions

This review has provided an overview of translational research and highlighted examples of translational research in the field of kidney transplantation. Translational research is an evolving discipline that emphasizes fluidity between the different phases of research and requires strong interdisciplinary collaboration among researchers and clinicians.

The example of histocompatibility testing, and how translational research has played a role in improving management options for sensitized individuals, demonstrates how focused efforts to bridge gaps between basic science research, clinical research, and implementation in clinical practice can lead to improved patient outcomes. Further, involving patients and stakeholders in establishing and monitoring research agendas increases the likelihood that research will be produced that is meaningful and relevant to patients and those who care for them. Potential rich areas for translational research efforts include non-invasive diagnostic test development for acute rejection, novel immunosuppression strategies, and improving medication adherence. Future use of established, transparent research prioritization initiatives involving key Canadian kidney transplantation stakeholders and evaluation of these processes will be not only important but also necessary to ensure that the concerns of research end-users are being addressed and that research findings are being implemented in practice. The future of translational research in kidney transplantation and beyond will require the deliberate fostering of partnerships to ensure that scientists, clinicians, and patients are working towards common goals.

## Abbreviations

CIHR: Canadian institutes of health research
ESRD: End-stage renal disease
mRNA: messenger RNA
NGAL: Neutrophil gelatinase associated lipocalin
ABMR: Antibody-mediated rejection
KDIGO: Kidney disease improving global outcomes
HLA: Human leukocyte antigen
MHC: Major histocompatibility complex
CDC: Complement-dependent cytotoxicity
PRA: Panel reactive antibody
PRO: Patient-reported outcomes
HRQoL: Health-related quality of life
